# Supplementary material for: Are carbon emissions trading and green financial instruments synergistic? -Comprehensive quantitative research based on content analysis
Source: PLoS One. 2024 Mar 7;19(3):e0298601. doi: 10.1371/journal.pone.0298601 (PMC10919601; doi:10.1371/journal.pone.0298601)
Supplement: S1 Data — (ZIP) [file pone.0298601.s001.zip › Data/地级市Z_F工作报告.zip!/╝¬┴╓/░╫│╟╩╨/└┤╫╘╣1⁄2╓┌║┼í╛┬φ┐╦╩2╛▌═°í┐_2019.docx]

各位代表：

　　现在，我代表市政府，向大会报告政府工作，请予审议，并请市政协各位委员提出意见。

　　一、2018年工作回顾

　　在改革开放走过40周年的关键时点，回望过去一年白城的发展，依然极不平凡。面对严峻复杂形势，我们在以习近平同志为核心的党中央坚强领导下，认真贯彻落实党中央国务院、省委省政府和市委的决策部署，团结带领全市干部群众，戮力同心、砥砺奋进，“三大攻坚战”完成阶段性任务，“四项重点工作”取得标志性成果，一系列重大考验化危为机、重大挑战有效应对、重大项目落地见效，开创了白城全面振兴发展的崭新局面。

　　（一）经济运行稳中有进，质量效益同步提升。落实高质量发展要求，着力稳增长、促转型，主要指标增速排在全省前列。预计全市地区生产总值增长5%，规上工业增加值增长6%，固定资产投资增长5%，总体呈现平稳向好态势。地方级财政收入增长6.8%，增幅全省最高。现代农业全面发展。主推10种新技术、新模式，棚膜面积发展到17.6万亩、增长27%。土地适度规模经营占比39%，耕种收综合机械化水平达到86%。洮南市被命名为中国绿豆特色农产品优势区、全程机械化示范县。农业生产能力的提升，加之降水充沛、雨热同期，全市粮食生产获得大丰收，总产量达到95.8亿斤，创历史最好水平；增长13%，居全省首位。有效防控非洲猪瘟、牲畜口蹄疫等动物疫病，畜禽规模化养殖比重接近50%。工业经济企稳回升。开展助企脱困活动，华锐风电恢复生产，德尔福派克新上全自动生产线，安费诺金事达产品达到欧6排放标准，雏鹰建成饲料智能化工厂，白城发电公司、中材科技、戈壁能源等50户重点企业产值增长20%以上。清洁能源装机增长14%，弃风率降至8%以下。全市新入统规上企业31户，新增省级科技小巨人企业、高新技术企业8户。工业投资增长94.9%，保持全省第一。服务业转型步伐加快。新增限额以上商贸流通企业41户。洮宝大学生创业园、森昊物流园获批省级现代服务业集聚区。电子商务交易额突破20亿元，外贸进出口增长8%。生态旅游发展势头良好，嫩江湾荣获“中国最美休闲胜地”称号。民营企业、个体工商户分别增长13.8%和7.4%，民营经济呈现蓬勃生机。

　　（二）项目攻坚再获突破，招大引强成效显著。按照全省“三抓”“三早”部署，深入开展精准谋划、精准招商、精准服务“三精准项目攻坚”。招商引资持续发力。坚持“产业树”理念，谋划包装亿元以上项目114个。成功举办白城—嘉兴对口合作商协会、燕麦产业发展合作对接会，九新30万吨燃料乙醇、鲁能15万千瓦风电开发、弘泰煤焦油轻质化二期等项目签约落地。项目建设加快推进。开复工5000万元以上项目203个，产业项目占比70%以上，创历史新高。工业园区“双创”中心、百琦中药配方颗粒、飞鹤乳品加工、东嘉垃圾焚烧发电等项目开工建设。中一精锻搬迁改造、澎派植物油、安广生物质热电联产等项目竣工投产。精准服务富有成效。落实重点项目秘书制，实行重大项目专班推进。东北地区唯一的50万千瓦光伏应用领跑基地，实现全容量并网发电，创造了优选评审、开工建设、并网发电等“七个全国第一”。尤为欣喜的是，投资100亿元的梅花集团300万吨玉米深加工项目，一期工程完成投资60亿元，实现当年开工、当年投产，不仅体现了负责任大企业超常的建设速度和一流的管理水平，更展现了白城干部服务大项目的高标高效和责任担当。

　　（三）环保督察整改有力，生态环境大幅改善。坚决打好污染防治攻坚战，加快建设生态白城、美丽白城。整改工作全面落实。以高度的政治自觉和行动自觉，狠抓中央和省生态环保督察反馈问题整改，整改任务全部达到序时进度。出台配套制度文件62项，治理保护长效机制更加健全。生态工程有序实施。完善河湖连通配套设施，引蓄水10.9亿立方米，国家水生态文明城市试点顺利通过验收。综合治理草原126万亩，规模化种植燕麦30万亩。植树造林37.5万亩，修复湿地24万亩，历时三年的“造林还湿双百万”任务超额完成。污染防治扎实开展。深化蓝天、清水、净土行动，我市荣获“吉林省节水型城市”称号。落实“一河一湖一策”，秸秆能源化利用、畜禽无害化处理、污水处理厂提标改造、盐碱地治理等绿色环保项目加快实施。在全省率先探索出市场化整合供热企业新模式，市区热电联产率达到85%，燃煤量、有害气体排放量均减少55%。空气优良天数占比97%，保持全省首位。持续的生态投入和有利的气候条件，使多年不见的湿地草原美景重现瀚海大地。

　　（四）精准扶贫扎实有效，脱贫战果持续扩大。聚焦“两不愁、三保障”，扎实开展精准脱贫攻坚战三年行动。产业扶贫发挥效益。推广“双带四增”产业扶贫模式，落实产业扶贫项目1442个。投入补贴资金1.4亿元，发展庭院经济19.6万户、18.8万亩。村级光伏扶贫电站运营良好，已给贫困户分红4664.6万元。电商服务站覆盖所有贫困村。扶贫车间发展到55个。政策保障精准落地。完善“三下沉两提高一兜底”医疗扶贫措施，支出政府资金4000余万元，惠及8万余人。“健康快车”项目圆满完成，免费为1207例贫困白内障患者实施复明手术。镇赉县健康扶贫工作获得国家卫健委、国务院扶贫办通报表彰。跟踪帮扶6196名贫困学生，没有一人因贫辍学。开展助行、助听、助视救助活动，1.5万名残疾人享受到精准康复服务。人居环境明显改善。改造危房9893户、厕所9114户，文化服务中心、健身路径、安全饮水实现贫困村全覆盖。通榆县完成31个村异地扶贫搬迁，大安市大岗子镇7个村实现整体搬迁。开展“六清”集中整治，村屯卫生保洁长效机制初步建立，成功打造17个市级样板，乡镇、村屯环境实现历史性改观。洮北区黎明村被评为“中国美丽休闲乡村”。包保帮扶深入开展。市县乡联动开展驻村“三帮扶一推动”活动，各县（市、区）相继开展“百日攻坚”“百日会战”等活动，“远学四川、近学镇赉”在全省形成共识。冬季大培训、百企助百村、驻军帮扶、志智双扶等活动富有成效。预计年度脱贫3.9万人、退出225个贫困村，镇赉县、洮南市通过脱贫摘帽初审，全市脱贫攻坚取得决定性进展。

　　（五）老城改造全面完工，海绵城市树立样板。坚持建设、管理接续发力，加快推进“四城联创”，打造最干净、最整洁城市。改造提升圆满收官。高质量完成了老城改造收尾工程，公园、广场、小区、步行街等设施配套完善，主要街路电力、通讯管线全部入廊，绿化、美化、亮化、净化全面提升，城市面貌华丽蜕变。海绵城市成效凸显。成功经受多次强降雨考验，实现大雨不内涝、小雨不积水。我市荣获全国海绵城市建设“样板城市”，创造了全面完工、经验总结、案例发行“三个全国第一”。全省城市老旧小区整治提升暨海绵城市建设现场会在我市召开。城市管理全面加强。开展“城市管理提升年”活动，“清洁城市•美丽家园”集中治理卓有成效。小区包保和物业管理实现全覆盖。智慧城市指挥中心投入使用。我市通过省级卫生城考核验收，被授予“吉林省园林城市”，获得“全国文明城市”提名。大安市、镇赉县、洮南市荣获省级文明城市。广大市民的文明素质、公德意识显著增强，获得感、幸福感、荣耀感前所未有。

　　（六）改革开放深入推进，营商环境全面优化。以改革开放40周年为重要契机，狠抓改革创新增动力，扩大开放添活力。重点领域改革不断深化。推动“三去一降一补”，减轻企业税费负担6亿元。电力直接交易5.8亿度，增长71%，有效降低用电大户成本。开展“金融助振兴”活动，对域内重点企业信贷投放17.6亿元。出台乡村振兴战略实施意见，土地确权颁证、国有林场改革、供销社综合改革试点、农业“两区”划定顺利完成。国有企业“三供一业”分离移交进度全省领先。“放管服”“只跑一次”改革步伐加快。发布新版政府部门权责清单，下放行政权力32项。公布“零上门”和“只跑一次”事项2006项，占比94.8%。“互联网+医保”典型做法获国务院通报表扬。社会信用体系建设全面加强，全国城市信用监测排名提升127位。我市荣获“中国企业营商环境十佳城市”。对外开放合作深度拓展。白齐兴跨省生态经济合作区列入国家《东北西部生态经济带规划》。我市与兴安盟签署区域合作协议，与长春市、嘉兴市对口协作取得积极进展。成功举办国家燕麦产业发展学术交流会、第二届道地关药产业发展论坛。平齐、通让铁路电气化改造竣工通车，双嫩、松通2条高速公路开工建设，国道302东绕越线全线贯通，白城机场运送旅客突破6万人次，日益完善的交通体系有力促进了开发开放。

　　（七）民生保障不断增强，社会大局和谐稳定。克服财政困难，优先解决群众关注的热点难点问题。民生实事全面兑现。新增城镇就业4.2万人，农村劳动力转移就业27.4万人。大学生、农民工返乡创业4960人。大安市荣获“全国农村创业创新典型县”。全市城镇常住居民人均可支配收入预计增长6%，农村常住居民人均可支配收入有望突破万元大关。提高医保、低保待遇标准，实现低保线、扶贫线“两线合一”。市养老服务中心开工建设，滑冰馆主体竣工，体育场投入使用。新能源公交枢纽投入运营，新建农村公路455公里，创建“四好农村路”示范路72条。推进“百兆乡村”项目，新增光纤用户1.8万户。棚改回迁安置1万套，公租房分配1500套。解决“无籍房”1万余户，完成进度居全省前列。社会事业协调发展。市农科院燕麦研发团队荣获“黄大年式科研团队”称号。镇赉县成来电气院士工作站获批，袁隆平院士工作站落户大安，柔性引进院士3人，院士团队专家45人。改造薄弱校19所，白城素质教育基地被评为“全国中小学生研学实践教育营地”。“健康白城”建设扎实推进，多层次医联体、医共体全面启动，创新完善肺结核患者保障体系在全国推广。在全省率先探索“互联网+家庭医生签约服务”新模式，实现了服务、互动“零距离”。开展庆祝改革开放40周年系列活动，我市被中宣部列入全国“百城百县百企”调研城市。通榆县被列入全国“新时代文明实践中心”建设试点。成功举办首届中国·白城半程马拉松。建成防震减灾科普馆，我市被评为“全国防震减灾工作先进单位”。军民融合深度发展，拥军优抚政策得到落实，驻地部队停止有偿服务如期完成。社会治理安定有序。全面加强政府监管，有效防范各类风险。深入开展“安全生产治理年”活动，安全生产形势稳中向好，未发生较大以上安全事故，实现食品、药品安全事件“双无”目标。化解信访积案410件，信访总量持续下降。扫黑除恶专项斗争深入推进，全国禁毒示范城市创建全面展开。民族宗教、外事侨务、广播电视、人口计生、妇女儿童、残疾人、老龄等工作取得新成效，气象、人防、档案、地方志、红十字会等事业实现新突破。

　　（八）作风整顿持续深入，自身建设全面加强。贯彻新时代党的建设总要求，推动政府系统党风廉政建设向纵深发展。政治建设不断强化。认真践行习近平新时代中国特色社会主义思想，深入贯彻习近平总书记对东北振兴工作的重要指示精神，积极推进“两学一做”学习教育常态化制度化，牢固树立“四个意识”，切实增强“四个自信”，坚决做到“两个维护”。依法行政加快推进。修订政府工作规则，制定常务会议工作规范。全面落实市人大、市政协视察调研提出的意见和建议，认真办理人大代表建议、政协提案，办复率达到100%。完成市政设施保护条例提请审议工作。开展法治政府建设专项检查，“双随机、一公开”综合监管实现常态化。作风建设扎实有效。严格落实中央八项规定精神，自觉接受监察监督，反腐倡廉工作取得新成效。认真开展干部作风大整顿、解放思想推动高质量发展大讨论活动，政府工作人员普遍经历了一次深刻的思想政治教育，解决了一批作风上的沉疴积弊，凝聚力、战斗力、执行力进一步增强。

　　各位代表！

　　奋斗充满艰辛，成绩来之不易。这是我们在以习近平同志为核心的党中央坚强领导下，认真贯彻党中央国务院大政方针，深入落实省委省政府和市委决策部署的结果，是人大、政协有效监督、大力支持的结果，是广大干部群众团结拼搏、奋发进取的结果，凝结了各方面的辛勤付出和聪明才智。我代表白城市人民政府，向全市各族人民，向各民主党派、工商联、人民团体、无党派人士、离退休老同志，向驻白中省直部门和企事业单位、人民解放军指战员、武警部队官兵、公安司法干警，向所有关心支持白城发展的各界朋友，致以崇高的敬意和衷心的感谢！

　　回顾过去一年，我们清醒地看到，在前进道路上还有许多困难和挑战，政府工作还有一些差距和不足。主要表现在：经济增长压力较大，具有支撑力、带动力的大项目不多，引增量、活存量、扩总量、提质量还需持续用力；生态环境依然脆弱，环保督察整改任务艰巨，水的瓶颈、盐碱地治理、秸秆综合利用等难点问题还没有从根本上破解，污染防治还需久久为功；扶贫基础工作还不够精准，有的产业扶贫项目未达到预期收益，有的政策宣传普及不到位，激发群众脱贫内生动力的办法不够多；城市文化元素欠缺、软实力不强，辐射带动能力亟待提升，农村环境整治水平参差不齐，农村生活垃圾和污水没有得到有效治理，城乡管理需要在精心精细、常态长效上创新举措；民生领域的短板仍然较多，就业、住房、城市安全、社会保障等水平不高，群众对医疗、教育、养老、食品安全等还有不满意的地方；营商环境有待进一步优化，公共服务存在薄弱环节，群众和企业办事还有“痛点”“堵点”，融资困难、负担较重、人才短缺等问题仍然突出；少数干部服务意识、法治意识不强，能力素质、工作作风不过硬，“四风”出现新动向新表现，一些重点领域的不正之风和腐败问题仍然多发。我们将坚持问题导向，以高度负责的精神、克难攻坚的意志，竭尽全力做好政府工作，奋力实现“一年一变化、三年大变化、五年上层次”，不负人民的期望和重托。

　　二、2019年工作安排

　　今年是新中国成立70周年，是全面建成小康社会关键之年。政府工作总的要求是：深入贯彻习近平新时代中国特色社会主义思想和党的十九大精神，坚持以习近平总书记对东北振兴工作重要指示精神为指引，统筹推进“五位一体”总体布局、协调推进“四个全面”战略布局，全面落实中央经济工作会议和省委十一届三次、四次全会、全省经济工作会议精神，按照市委六届五次全会部署，紧紧围绕实现高质量发展主题、建设吉林西部生态经济区主线，坚持推进科学发展、坚持深化改革开放、坚持保障改善民生，大力实施“三个五”发展战略和“一主六双”产业空间布局，继续打好“三大攻坚战”，突出抓好“四项重点工作”，加快实现各项事业新突破，奋力推进白城全面振兴、全方位振兴，为全面建成小康社会、让全市人民过上更加美好的日子不懈奋斗。

　　按照中央“六个稳”工作要求，坚持引进增量、盘活存量、做大总量、提高质量“四量并举”，今年全市经济社会发展的主要预期目标是：地区生产总值增长6%；地方级财政收入增长3%；固定资产投资增长7%；规上工业增加值增长7%；社会消费品零售总额增长5%；城乡常住居民人均可支配收入与经济增长基本同步；城镇新增就业2.1万人，登记失业率控制在4.5%以内；节能减排指标完成省下达任务。

　　着力做好以下十个方面工作：

　　（一）聚焦美丽白城，持续加强生态建设。深入实施生态保护与建设五年行动计划，加快构建生态环境治理、生态投入保障、生态文明制度“三大体系”，着力提升生态文明建设水平。一是狠抓环保督察整改。坚持“一案一策”、盯紧压实，实行台账管理、跟踪督办，确保所有整改任务按时销号、所有信访案件限时整改到位。注重举一反三，健全长效机制，坚决防止同一问题反复、同类问题反弹。完善市县两级长期举报电话受理机制，做到凡举必查、查实必改、监管必严。二是推进水草林湿治理。实施区域水循环利用、草原生态治理、三年再造百万林、湿地保护修复“四大工程”，推进河湖连通达标、引嫩入洮、农田林网改造、绿色通道建设、集中连片万亩草场建设等项目，新增水面50万亩，恢复草原175万亩，植树造林35万亩，修复湿地50万亩。三是加强环境污染防治。坚决打好污染防治攻坚战，精准落实清洁空气、水体、土壤行动计划。大力推进秸秆“五化”综合利用。完成建成区燃煤小锅炉淘汰改造。全面整治河湖乱占、乱采、乱堆、乱建问题。抓好餐厨剩余物、畜禽粪污资源化利用。推进化肥农药使用量零增长。四是健全生态保护制度。深化河长制、湖长制，探索实行林（草、湿）长制，完善生态建设目标责任、考核评价、执法监督等制度。加强资源环境生态红线管控，建设天蓝、地绿、水清的美丽白城。

　　（二）紧扣精准脱贫，扎实推进生态扶贫。严格落实“六个精准”，突出“三个巩固提升”，对照户脱贫、村退出、县摘帽标准，持续扩大脱贫成果。一是强化产业扶贫。加大资金整合力度，再落实一批规模化扶贫大项目。加强村级光伏电站运营管护，完善收益分配机制。推动庭院经济规模化、特色化、品牌化发展，与电商平台互联互通，让小庭院成为“增收园”。实施乡村小微企业培育计划，新建扶贫车间33个。建立扶贫产业风险补偿机制，保障扶贫产业稳定发展、贫困人口稳定脱贫。强化扶贫资金资产管理，确保资金安全高效、资产保值增值。二是落实兜底政策。进一步抓好现有保障性政策落实，继续在医疗、教育、低保、助残等方面查遗补漏，解决好特殊群体和特殊事例。针对收入水平略高于建档立卡贫困户的群体，研究解决缺乏政策支持等新问题。出台《关于县（市、区）摘帽后扶贫成效巩固提升的意见》，保持扶贫政策不变、投入力度不减。三是加强环境整治。深入开展农村人居环境整治三年行动，启动村庄清洁行动，重点抓好农村生活垃圾、建制镇污水处理、厕所革命、村容村貌提升，同步完善基础设施、公共服务设施。开展爱国卫生运动，建立“六清”长效保洁机制，从根本上解决脏乱差问题，所有的村都要达到干净整洁标准。四是深化志智双扶。持续开展“三帮扶一推动”活动，注重解决实际问题，提高群众认可度和满意率。大力宣传企业、驻军、个人和社会组织帮扶的有效形式，打造扶贫公益品牌。开展志智双扶“十佳五星”评选，加强就业政策、劳动技能、实用技术培训，让群众自主脱贫有愿望、有路子、有能力。通过全市上下共同努力，确保绝对贫困人口全部脱贫、贫困村全部退出、贫困县全部摘帽。

　　（三）突出项目带动，再掀招商引资热潮。深入落实“三抓”“三早”部署，实施重点项目三年计划，开展好“项目建设年”活动。一是提高谋划精度。抢抓政策机遇，聚焦培育新动能、打造产业链，突出农畜产品精深加工、清洁能源本地消纳、生态旅游资源开发，谋划重点项目200个以上，工业项目占比50%以上。二是加大招商力度。做好“产业树”招商规划，举办氢能经济、燕麦产业等专题招商。实施“鹤商回巢”行动，结合开展“全国媒体进白城”“白城名人回家乡看白城”活动，加大招商引资、项目推介力度。深耕在谈企业，跟踪推动79个在谈项目尽早落地。各地至少引进2个投资超10亿元的工业项目。三是加快建设进度。落实领导包保和项目企业“专员制”，完善项目生成、落地、推进机制。实施超亿元项目100个以上，工业投资占比60%以上。重点抓好梅花集团300万吨玉米深加工二期、益海嘉里34万吨稻米深加工、九新30万吨燃料乙醇、俄罗斯天然气“松白乌”管道等项目开工建设，推动工业园区“双创”中心、百琦中药配方颗粒、飞鹤乳品加工、洮南香酒业搬迁改造、吉运15万头肉牛、牧原200万头生猪等项目竣工投产，为稳增长注入强劲动力。

　　（四）强化转型升级，加快壮大生态工业。按照“三三一产业”发展规划，深入实施小微企业生成、重点企业培育、重点产业跃升“三大计划”，确保工业经济稳健增长。一是产业发展突出抓好“双谷双基地”建设。落实国家级高载能高技术基地建设规划，围绕打造中国北方云谷，启动华为数据中心、吉林移动灾备中心、中航科技数据中心等项目，推动联通云计算中心扩容，加快建设数字白城；围绕打造中国北方氢谷，委托中国工程院等高端智库编制专项规划，快速推进风电制氢、加氢站等项目建设，加快构建制氢、储氢、运氢、用氢和氢能装备全链条产业集群；围绕打造新能源示范基地，再建50万千瓦光伏应用领跑基地，重新启动通榆230万千瓦风电基地建设，实施鲁固直流风电开发、生物质发电项目，新增新能源装机100万千瓦以上；围绕打造高载能产业绿色发展基地，推进通威集团光伏组件、三一风电装备制造等项目，加快推动制造业高质量发展。二是企业培育突出加大民营经济扶持力度。出台深入推进民营经济发展的实施意见，全面落实减税降费政策，完善融资、创新、市场、创业、人才“五大服务体系”。开展法律服务，保护企业家人身和财产安全，让企业家放手发展、安心创业。践行亲清新型政商关系，常态化开展领导干部帮扶企业活动。支持“个转企”200户，力争“小升规”20户以上，科技小巨人企业、高新技术企业发展到30户，培育更多优质企业。充分发挥企业家协会、商会作用，建设优秀企业家队伍。三是平台建设突出开发区改革创新发展。优化功能布局，完善管理体制。依法清理僵尸企业，通过腾笼换鸟，尽快盘活闲置资源。建设燕麦、中医药、绿色化工等特色产业园，提升产业集中度、竞争力。加快壮大白城经开区、工业园区综合实力。推动洮南开发区、洮北开发区晋升省级开发区，全面增强各开发区承载力，打造县域经济发展的增长极。

　　（五）实施乡村振兴，大力发展生态农业。出台乡村振兴战略五年发展规划，推动“三农”工作再上新台阶。一是加快农业产业化。实施农牧业高质量发展“五大行动”。培育合作社示范社200个、成长型龙头企业20户、种养加小微企业100户。开展国家级特色农产品优势区、农产品质量安全示范县创建，启动玉米、水稻、杂粮杂豆等5个绿色高质高效示范区建设。扎实推进“大棚房”问题专项清理整治。狠抓重大动物疫病防控，发挥无疫区优势。实施数字农业创新工程，扶持建设10家“云农场”，探索农产品“私人订制”、农业生产可视可控可追溯。开展首届农牧业名片价值品牌评选，打好“白城大米”“白城燕麦”“白城绿豆”“白城甘草”等特色品牌。推进雏鹰、吉运、吉天然等三产融合项目，开展国家级现代农业产业园创建，加快传统农业向现代食品工业转变，提升产业链水平。二是促进农民增收。发展高效避灾农业，聚集龙头企业、工商资本，建设市级以上规模化棚膜园区20个，促进四季增收。大力推广新技术新模式，促进提质增收。全面落实粮食安全责任制，推进“粮安工程”，促进减损增收。鼓励龙头企业、合作社、家庭农场适度规模经营，推行土地股份合作、托管，发展订单种植、订单养殖，促进带户增收。积极发展休闲农业、乡村旅游，打造特色产业示范镇5个、特色村20个，促进多元增收。三是强化乡风文明和乡村治理。抓好“新时代文明实践中心”、文化大院等建设，开展移风易俗，搞好星级文明户评比，培育文明乡风、良好家风、淳朴民风。坚持自治、法治、德治相结合，健全村规民约，打造充满活力、和谐有序的善治乡村。

　　（六）发展现代服务业，着力培育生态旅游。注重发挥优势、打造特色品牌，推动服务业转型提质。一是打造全域旅游。推进军事体验基地、影视基地、仿古一条街、冰雪旅游、红色旅游等项目，加强资源整合，塑造精品景点。积极开发研学游、自驾游线路，构建河湖草原湿地旅游大环线。筹划举办首届旅游文化节、国际摄影展系列活动，大力推介白城旅游的特色和魅力。二是提升商贸流通。支持零售企业创新经营模式、组织形式，引导发展特色商业街、商业综合体。推动便利店、社区超市拓展便民增值服务，打造“一刻钟”便民生活服务圈。启动建设会展中心，创建全国信息消费试点城市。三是做大现代物流。抓好中农冷链物流交易中心、大安物流产业园等项目建设，确保国检中心投入运营。加快推进“快递下乡”，全面提升电商服务能力，鼓励企业发展跨境电商，力争进出口总额实现翻番。同时，顺应消费转型升级新趋势，大力发展住房租赁市场，促进文化体育、健康养老等领域消费提质扩容，形成新的经济增长点。

　　（七）注重品质提升，倾力打造生态城市。坚持高标准定位、高起点规划、高品质建设、高效能治理，全力推进“四城联创”，巩固提升区域中心城市带动能力。一是完善城市功能。启动城市空间规划编制工作，全面推进“多规合一”。谋划打造运河带状公园、鹤鸣湖、天鹅湖、东湖、青山草场“一带三湖一场”环城生态景观，构建“多点支撑、生态融城”空间格局。实施污水管网提质增效改造、道路照明节能改造等“十大基础工程”，推进五星级宾馆建设，强化综合承载能力。二是提升城市品位。谋划新建体育馆，合理布局城市阅读驿站和楹联文化街，筹办国际性草原音乐节，着力培育城市“软实力”，打造“书香白城”“音乐白城”“文化白城”。加快县城改造步伐，建设有品位、有活力、有意境的生态宜居县城。三是狠抓城市管理。扎实开展城市管理效能提升三年行动，抓好海绵城市、管廊城市运营维护，深化“门前三包”、垃圾分类等专项整治，启动创建国家级卫生城申报工作。开展城市绿化、市民文明行为促进等方面立法工作。全面完成城市管理执法体制改革，推动城市管理重心下移、职责下沉。创新“城管+大数据”模式，加强精细化管理，力争用两年时间达到全国一流水平。四是培育城市文明。持续推进文明城创建“八大攻坚行动”，开展文明素质提升“六大活动”，抓好文明单位、文明村镇、文明家庭、文明校园等群众性精神文明常态化创建，必保2020年步入“全国文明城市”行列。

　　（八）深化改革开放，全面优化营商环境。聚焦突出难题，谋求破解良方，进一步释放发展动力和活力。一是推进“放管服”“只跑一次”改革。持续简政放权，赋予县（市、区）和开发区（园区）更多自主权。全面优化政务服务，加大“一网、一门、一次”工作力度，完善政务大厅功能和设施，着力提升行政审批效能、提高网上办事比例，确保“零上门”和“只跑一次”事项不断增加。强化事中事后监管，推行“双随机、一公开”。全面抓好社会信用体系建设，完善联合激励惩戒机制，打造公平开放、透明高效、诚实守信的法治化营商环境。二是推进重点领域改革。深化供给侧结构性改革，在巩固、增强、提升、畅通上下功夫。落实“三去一降一补”任务，降低各类营商成本，加大基础设施等领域补短板力度。推进农村土地“三权分置”、集体产权、供销社等改革。健全地方金融、政府债务监管机制，坚决打好防范化解重大风险攻坚战。抓好国家级农村金融综合改革试验，实施金融集聚、上市驱动工程。研究筹建城市商业银行、地方产业投资基金，力争三年内引进2家国内大型股份制商业银行。完成国有企业“三供一业”分离移交，抓好国有资本投资、运营公司改革。结合群众的现实需求，针对社会保障等民生领域问题，推出一批改革举措。全面完成政府机构改革。统筹实施财税体制、生态文明等方面改革。三是推进全方位对外开放。深度融入“一带一路”，狠抓“五个合作”，吸引域外优势资本、先进技术、优秀人才向白城汇聚。加快构建白齐兴跨省生态经济合作区，争取作为《东北西部生态经济带》先导区，从国家战略层面实施推进。密切对接长春市、嘉兴市，力争在土地指标交易、承接产业转移、共建产业园区、开发旅游市场等方面取得新突破。加快推进双嫩、松通高速建设，改造升级扎赉特旗至突泉公路，争取开通机场新航线，积极推进快铁提速，以大交通促进大流通、以大流通促进大开放。

　　（九）保障改善民生，维护社会和谐稳定。突出保基本、兜底线，更加关注补齐民生短板，让人民群众共享振兴成果。一是扎实办好民生实事。主要有十个方面：（1）实施创业就业工程，举办青年创业创新大赛，创建省级农民工返乡创业基地2个。农村劳动力转移就业27万人，城镇零就业家庭援助率100%。（2）深入推进全民参保计划，基本医疗保险率达到100%。提高城乡低保、退休人员基本养老金、工伤人员待遇标准。（3）抓好保障性安居工程，棚改回迁安置8000套，力争两年内消灭全部棚户区。（4）实施“关爱困难群体”行动，对城市低保中重病、重残、高龄失能、单亲家庭学生等特殊困难群体，加大救助帮扶力度。探索建立特困失能人员集中供养机制。（5）实施幸福养老工程，市居家养老服务示范中心投入使用，每个县（市、区）确保有1所医养结合服务机构，推进乡镇福利养老中心整合，提升居家养老服务水平。（6）开展残疾人精准救助活动，为1万名有需求的残疾儿童和持证残疾人提供基本康复服务，为580户贫困残疾家庭进行无障碍改造。（7）实施文体惠民工程，持续开展“歌舞鹤乡•放飞梦想”群众文化系列活动，举办第二届中国·白城马拉松，建设农村健身路径100个，市游泳馆投入使用。农村光纤再覆盖3.6万户。（8）推进新一轮改善医疗服务行动计划，落实国家级“全民健康定点帮扶援助项目”，确保重点人群家庭医生签约率达到75%。（9）实施城乡交通改善工程，开工建设城区西部客运换乘中心、平齐铁路3处公铁立交桥，建设“四好农村路”200公里。（10）创建“智慧民生”服务平台，设立热线电话，实现“一个电话解难题”。二是全面发展社会事业。深入实施“人才兴市”战略，全面落实住房、家属就业、子女教育等相关待遇，着力引才、育才、留才。抓好考试招生制度改革，优化城乡教育资源配置。设立中高职重点企业冠名班、订单班，筹建陈香美院士工作站。谋划建设文化产业创业园，培育文化创意企业。引进高水平专科医院，启动创建三级甲等医院，抓好母婴健康项目落实。持续强化军民融合发展，加强国防后备力量建设。广泛开展拥军优属、拥政爱民活动，做好新一轮全国双拥模范城申报工作。编纂《知白城爱家乡》方志读物。抓好第三次国土调查、第四次经济普查工作。全面深入开展民族团结进步创建工作，大力发展公益慈善、妇女儿童、退役军人等事业，扎实做好外事侨务、宗教、人防、气象、地震等工作。三是创新加强社会治理。建设“大安全”体系，创建平安白城。借鉴推广“枫桥经验”，创新矛盾调解机制，推进基层社会治理现代化。深入开展扫黑除恶专项斗争，促进扫黑除恶与打伞治乱紧密衔接，推动由打击遏制向深挖根治迈进。抓好“警务云”建设应用，力争三年内视频监控覆盖所有村屯，打造全省最具安全感城市。落实党政领导干部安全生产责任制《规定》《实施细则》，推进“五个一工程”，巩固提升“四化融合”。依法推动信访问题及时就地解决，每个县（市、区）必保再化解50件信访积案。坚决落实“四个最严”要求，加强食品药品监管。着力提高自然灾害防治能力，让人民安居乐业、社会安定和谐。

　　（十）强化作风转变，建设人民满意政府。各级政府必须全面加强自身建设，彰显新气象、展示新担当、体现新作为。一是加强政治建设。落实全面从严治党要求，严守政治纪律和政治规矩。认真开展“不忘初心、牢记使命”主题教育，进一步增强“四个意识”，坚定“四个自信”，坚决维护习近平总书记核心地位，坚决维护党中央权威和集中统一领导。二是加强法治建设。带头遵守宪法和法律，完善法律顾问制度。依法接受人大及其常委会监督，自觉接受政协民主监督，提高建议、提案办理质量。加大政务公开力度，主动接受社会和舆论监督。全面推进法治政府建设，深入实施“七五”普法，提高依法治市水平。三是加强作风建设。坚决贯彻中央八项规定精神，驰而不息纠“四风”、破“五弊”，力戒形式主义、官僚主义新表现。改进调查研究，深入解放思想。整治文山会海、规范督查检查，把干部从一些无谓的事务中解脱出来。坚持严管和厚爱结合、激励和约束并重，给谋事干事成事者撑腰鼓劲。四是加强廉政建设。认真履行政府党组主体责任，严格落实“一岗双责”。强化审计监督，主动接受监察监督。加强对扶贫、招投标、政府采购、国企国资等重点领域监管，持续保持惩治腐败高压态势。政府工作人员要进一步提高执行力，以良好的形象、出色的业绩，来赢得人民群众的好赞誉、好口碑。

　　各位代表！

　　团结凝聚力量，实干铸就辉煌。让我们紧密团结在以习近平同志为核心的党中央周围，在习近平新时代中国特色社会主义思想的科学指引下，在省委、省政府和市委的坚强领导下，锐意进取、迎难而上，推动白城高质量发展、全方位振兴，为全面建成小康社会、让全市人民过上更加美好的日子而拼搏奋斗，以优异成绩向新中国成立70周年献礼！
